# TagCleaner: Identification and removal of tag sequences from genomic and metagenomic datasets

**DOI:** 10.1186/1471-2105-11-341

**Published:** 2010-06-23

**Authors:** Robert Schmieder, Yan Wei Lim, Forest Rohwer, Robert Edwards

**Affiliations:** 1 Department of Computer Science, San Diego State University, San Diego, CA, USA; 2 Computational Science Research Center, San Diego State University, San Diego, CA, USA; 3 Department of Biology, San Diego State University, San Diego, CA, USA; 4 Mathematics and Computer Science Division, Argonne National Laboratory, Argonne, IL, USA

## Abstract

**Background:**

Sequencing metagenomes that were pre-amplified with primer-based methods requires the removal of the additional tag sequences from the datasets. The sequenced reads can contain deletions or insertions due to sequencing limitations, and the primer sequence may contain ambiguous bases. Furthermore, the tag sequence may be unavailable or incorrectly reported. Because of the potential for downstream inaccuracies introduced by unwanted sequence contaminations, it is important to use reliable tools for pre-processing sequence data.

**Results:**

TagCleaner is a web application developed to automatically identify and remove known or unknown tag sequences allowing insertions and deletions in the dataset. TagCleaner is designed to filter the trimmed reads for duplicates, short reads, and reads with high rates of ambiguous sequences. An additional screening for and splitting of fragment-to-fragment concatenations that gave rise to artificial concatenated sequences can increase the quality of the dataset. Users may modify the different filter parameters according to their own preferences.

**Conclusions:**

TagCleaner is a publicly available web application that is able to automatically detect and efficiently remove tag sequences from metagenomic datasets. It is easily configurable and provides a user-friendly interface. The interactive web interface facilitates export functionality for subsequent data processing, and is available at http://edwards.sdsu.edu/tagcleaner.

## Background

Scientific interest in environmental microbial and viral communities is growing with every year. Metagenomics is an approach widely used to characterize microbial and viral communities for ecological studies and viral discovery across a wide range of environments such as marine, insects, plants, animals, and human [1-4]. The methodologies for metagenomic studies have been developed and refined throughout the years based on the characteristics of the samples. However, the methodology for characterizing RNA viral communities remains challenging, especially from small volume biological samples such as blood plasma, swab samples, and tissue biopsy, due to limited quantity and quality of the sample, as well as the low number of viral particles in these samples.

A typical metagenomic approach starts from the purification of viral particles coupled with the removal of the host and environmental materials, followed by viral nucleic acid extraction, sequence-independent amplification, and sequencing [5]. Metagenomic sequences can be generated in high quantities using next-generation high-throughput sequencing technologies such as the Genome Sequencer FLX system (Roche, Branford, CT). The immense amount of metagenomic data produced today requires an automated approach for data processing and analysis. Major steps of a typical sequence processing pipeline include sequence cleaning, fragment assembly, clustering, taxonomic assignment, and estimation of the community composition. The sequence cleaning step is an essential first step of the sequence processing pipeline before any further data processing in order to allow accurate downstream analysis. For metagenomic datasets, the sequence cleaning step usually includes filtering of duplicated reads, short reads, low quality reads, contaminations, and reads containing ambiguous bases (N) above a certain threshold.

Generating RNA viral-associated metagenomes may require the use of reverse transcriptase-mediated cDNA library synthesis, which generates the DNA template for sequencing. The Transplex Whole Transcriptome Amplification (WTA) approach (Sigma-Aldrich, St Louis, MO) was used to generate our RNA viral-associated metagenomes. This method is based on theoretical random PCR amplification using PCR primers with a random nucleotide sequence at the 3'-end and a defined sequence at the 5'-end [6,7]. Transplex WTA utilizes non-self complementary primers comprising a quasi-random 3'-end and a universal 5'-end in generating the cDNA library. This set of primers allows the elimination of 3'-bias, maximum amplification efficiency and the maintenance of representation during cDNA library amplification. PCR amplification using primers complimentary to the universal 5'-sequences is then performed to generate enough nucleic acids (approximately 2 to 3 *μ*g) for subsequent applications, such as sequencing.

The processing of metagenomic datasets generated from primer-based amplification such as the WTA method requires an additional step for sequence cleaning - trimming of the primer sequences. For the purpose of this article, any such artifacts at the end of the reads will be referred to as *tag sequences*. Algorithms for string matching that allow errors (also known as approximate string matching) can be used to account for sequencing errors. The approximate string matching problem is to find substrings that match the query with *k *or fewer errors. An error model is used to define how different two strings are. One of the most widely used error models is the so-called *edit distance*, which allows deleting, inserting and substituting characters in both strings. If all the operations have cost 1, *simple edit distance *is the minimum number of insertions, deletions and substitutions to make both strings equal. In the case of matching a tag sequence to a sequence read, the simple edit distance should transform the tag sequence into a subsequence of the read (ignoring end gaps extending the tag sequence to the length of the read). This study is focused on online searching for approximate string matching, which is different from indexed searching. Indexed searching requires the process of building a persistent data structure (an index) on the data to speed up the search later [8]. However, the single search on a sequence dataset for tag removal does not justify the extra space and time that is required for generating the index. Furthermore, indexed approximate string searching is a much more difficult problem and not as well studied as online approximate string matching.

Many algorithms have been developed during the last 50 to 60 years in the fields of signal processing, text retrieval and computational biology. Due to the large amount of literature, the reader will be referred to [9] for a good reference on approximate string matching applications for computational biology. A new era for approximate string matching was started in the 1990's by exploiting computational parallelism. The basic idea of parallelizing an algorithm using bits was introduced by Baeza-Yates [10]. Using the bit-parallelism, the number of operations that an algorithm performs can be reduced by a factor of at most the number of bits in a computer word. This speedup can be significant considering existing architectures with 64 bits. There are different approaches on how to parallelize the algorithms. Approximate string matching algorithms can parallelize the work of the dynamic programming matrix as described by Myers in [11]. Myers' algorithm represents the differences along columns of the dynamic programming matrix instead of the columns themselves, requiring only two bits per matrix cell. The current values of differences can be represented using binary vectors. A logical rather than an arithmetical approach as used in [12] allows updating the vectors in a single operation. The result is an approximate string matching algorithm with a worst case of *O*(*nm/w*), where *n *is the length of the text, *m *the length of the query and *w *the word size of the machine [11]. This algorithm for the general string matching case was adapted to process biological sequence data.

The trimming of the tag sequence is not trivial. Sequencing approaches such as pyrosequencing as implemented by Roche's 454 technology have their limitations. Base repeats, for example, might not be correctly identified due to noise in the flowgrams and can therefore generate sequences with variable tag sequences. The major source of noise is that the light intensities may not correctly reflect the homopolymer lengths and therefore result in either deletions or insertions [13-15]. To use an example from a real dataset: the true tag sequence is GTG GTG TGT T**GG G**TG TG**T TT**G G, not including the random nucleotides at the 3'-end. Instead, GTG GTG TGT T**GG **TGT G**TT **GG was observed, a tag sequence with two deletions (both in the nucleotide triplets). Insertions and deletions were identified in every tag-labeled metagenomic dataset examined. In three example libraries, less than 90% of the sequences have the correct 5'-end tag sequence, whereas more than 9% contained one or two insertions and/or deletions (Table 1). Algorithms such as PyroNoise [16] try to account for the noise in flowgrams, but require the raw flowgram data that is not always available to the end-user.

**Table 1:** Results for exact and approximate tag sequence matching

Tag sequence	Library	Reads matching with # Mismatches						
		**0**	**1**	**2**	**3**	**4**	**5**	**> 5**

5'-end	LIB019	38,271 (89.37)	2,253 (5.26)	564 (1.32)	185 (0.43)	50 (0.12)	64 (0.15)	1,438 (3.36)

	LIB020	14,491 (84.60)	1,629 (9.51)	430 (2.51)	165 (0.96)	31 (0.18)	24 (0.14)	359 (2.10)

	LIB021	41,764 (84.74)	4,748 (9.63)	1,345 (2.73)	427 (0.87)	125 (0.25)	111 (0.23)	762 (1.55)

3'-end	LIB019	7,194 (16.80)	12,156 (28.39)	2,454 (5.73)	688 (1.61)	683 (1.59)	766 (1.79)	18,884 (44.10)

	LIB020	2,855 (16.67)	2,460 (14.36)	561 (3.28)	279 (1.63)	275 (1.61)	904 (5.28)	9,795 (57.18)

	LIB021	7,981 (16.19)	6,924 (14.05)	1,800 (3.65)	942 (1.91)	908 (1.84)	2,480 (5.03)	28,247 (57.32)

Concatenated	LIB019	931 (2.17)	282 (0.66)	132 (0.31)	51 (0.12)	104 (0.24)	32 (0.07)	-

	LIB020	185 (1.08)	45 (0.26)	19 (0.11)	12 (0.07)	17 (0.10)	8 (0.05)	-

	LIB021	1,302 (2.64)	464 (0.94)	215 (0.44)	120 (0.24)	135 (0.27)	30 (0.06)	-

Results for the 5'-end tag sequence (5'-GTG GTG TGT TGG GTG TGT TTG GNN NNN NNN N; Length: 31 bp; matching within 46 bp), 3'-end tag sequence (NNN NNN NNN CCA AAC ACA CCC AAC ACA CCA-3'; Length: 30 bp; matching within 45 bp) and the concatenated tag sequences (Length: 61 bp). Note that the numbers are based on the dereplicated datasets. Percentages are shown in parenthesis.

Tag sequences, especially WTA primer sequences, may contain ambiguous or random bases used for the sequence-independent amplification. This requires an approximate string matching algorithm that can be extended from the International Union of Pure and Applied Chemistry (IUPAC) ambiguity code for nucleic acids to define and identify the correct tag sequence in the query data. Myers' bit-vector algorithm can be easily extended with wildcard characters for the use of approximate DNA sequence matching.

**Figure 1:**
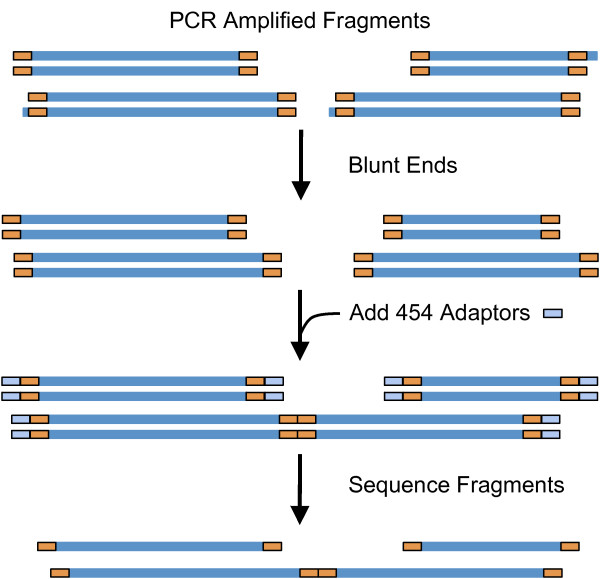
**Simplified model showing how fragment-to-fragment concatenations can be generated**. DNA polymerase can create overhangs during PCR amplification. An overhang is a stretch of unpaired nucleotides in the end of a DNA molecule (e.g. a single adenosine as a 3'-overhang). The unpaired nucleotides are removed to generate blunt-ended DNA molecules with both strands terminating in a base pair. This step can produce fragment-to-fragment concatenations because blunt ends are compatible with each other. The 454 adaptors are added to the amplified fragments by blunt-end ligation before sequencing. The resulting sequence data can contain artificial concatenated sequences.

The algorithm implemented here was also optimized to reflect the nuances of the sequencing approach in general, and the WTA approach in particular. The 454 adaptors are added to the WTA-amplified fragments by blunt-end ligation (see standard manufacturer protocol; section "General Library Preparation"). This step can produce fragment-to-fragment concatenations that give rise to artificial concatenated sequences (Figure 1). The resulting reads may contain concatenated sequence tags of more than 60 bp in addition to the sequence tags at the ends of the reads. Further analysis of such datasets may, for example, result in incorrect assemblies of the sequences or incorrect taxonomic assignments.

Fragment-to-fragment concatenations have been identified in every metagenomic dataset examined and occurred on average more than 2% of the time (Table 1). These fragment-to-fragment concatenations can be computationally identified and split, generating at least two separate sequences.

In addition, the length of the fragment from WTA-amplified cDNA may vary from 100 bp to over 1,000 bp (see Additional file 1). Current high-throughput sequencing methods such as pyrosequencing can generate sequence reads in the range from less than 100 bp up to 800 bp (500 bp average). The difference in fragment length and possible sequencing length may result in incomplete sequences that contain only part of the tag sequence at the 3'-end while some may contain no 3'-tag sequence at all. In order to account for this, the algorithm must accommodate differential trimming parameters at the 5'-end and 3'-end of the sequencing reads. The identification and removal of tag sequences in the dataset requires the *a priori *knowledge of the tag sequence used in the experiment. This information is often omitted from public databases or not available to the user due to, for example, patented methods. Our implementation therefore includes a feature for automatic tag sequence detection based on the nucleotide frequencies at the ends of the reads.

## Methods

### Sample collection and metagenomic library preparation

Coxsackie virus infected mouse brain tissues were homogenized and DNase treated prior to RNA extraction using Trizol-LS (Ambion, Austin, TX). Mosquitoes (*n *= 450) were collected from the San Diego Zoo Wild Animal Park in April 2009 using CO_2 _baited CDC traps (BioQuip Products, Inc., Rancho Dominguez, CA). The mosquitoes were pooled and homogenized in suspension medium (SM) buffer (100 mM NaCl, 10 mM MgSO_4_. 50 mM Tris-HCl pH 7.4). Samples were filtered through 0.45 *μ*M (Millipore, Billerica, MA) to remove large particles, followed by DNase treatment and RNA extraction using Trizol-LS (Ambion, Austin, TX). RNA was amplified using the Transplex Whole Transcriptome Amplification kit (Sigma-Aldrich, St Louis, MO). WTA-amplified cDNA libraries were then used for the Genome Sequencer FLX systems sequencing library preparation. Double-stranded cDNA was treated as sonicated DNA and proceeded directly to fragment size selection using the titrated amount of Agencourt AMPure SPRI beads (Agencourt Bioscience Corp., Beverly, MA). The ends of the fragments were polished and ligated with 454 adaptors prior to emulsion PCR as recommended by the manufacturer's protocol (454 GS FLX General Library Preparation Method). The 454 multiplex adaptors were generated according to the manufacturer's protocol (454 Technical Bulletin No. 004-2009) and used for all libraries. The amplified material was sequenced in-house with the Genome Sequencer FLX pyrosequencing system (Roche, Branford, CT) using the Titanium chemistry. The three viral metagenomes are accessible from NCBI http://www.ncbi.nlm.nih.gov under the genome project ID 46359.

### Sequence read preprocessing

Multiplexed reads were separated according to their MID tags (Table 2) and stored in FASTA format. TagCleaner was used to trim off the MID tags from the 5'-ends and to dereplicate the datasets (remove exact sequence copies). Sequence reads without an exact matching MID tag were excluded from this study.

**Table 2:** Datasets generated for and analyzed in this study.

Source	Library	# Reads	# Dereplicated	# Bases	Average length	# Reads with N's	MID tag	Data ID
Mosquito	LIB019	47,299	42,825 (90.54)	10,671,175	249.18	2,539 (5.93)	ATCAGACACG	31323732353938323030

Mouse	LIB020	18,620	17,129 (91.99)	3,164,017	184.72	3,273 (19.11)	ATATCGCGAG	31323732353938323133

Mouse	LIB021	53,062	49,282 (92.88)	9,428,045	191.31	9,639 (19.56)	CGTGTCTCTA	31323732353938323631

The data ID can be used to access the results in the TagCleaner web interface. Percentages are shown in parenthesis.

### Adapting the bit-vector algorithm for approximate tag sequence matching

The bit-parallel algorithm of the dynamic programming matrix was described by Myers [11]. The algorithm was extended with wildcard characters as described below. For our purposes and for completeness, some notations are introduced to show the changes made to Myers' algorithm.

Let *w *be the length of a computer word (in bits; e.g. 32 or 64). Let ∑ be a finite alphabet of the letters A, C, G, T and N. A string *s *is an ordered array of letters drawn from ∑. Let *s*_1 _be the query sequence and *s*_2 _be the tag sequence. Let *n *be the length of *s*_1 _and *m *≤ *w *be the length of *s*_2_. *ed*(*s*_1_, *s*_2_) denotes the edit distance between strings *s*_1 _and *s*_2_, which measures the minimum number of edit operations to transform *s*1 into *s*_2 _(and vice versa), ignoring end gaps. Given a pair of strings and a threshold *T*, any edit operation is called a mismatch between the two sequences, and a sequence does not match another sequence if the number of mismatches is greater than *T*.

The tag sequence *s*_2 _is expected to be located at the ends of *s*_1_. In the first search step, a subsequence  of *s*_1 _from 0 to max {10, 3*m*/2} is used to match *s*_2 _using the bit-parallel implementation. The algorithm is stopped at any iteration if a perfect match with *ed*(, *s*_2_) = 0 is found. The algorithm continues to search in the remaining of *s*_1 _to identify tag sequence repeats and fragment-to-fragment concatenations.

### Ambiguity code extension

The bit-vector algorithm was extended with wildcard characters as described in [11]. A wildcard character is a character that can be substituted for any other character of a defined subset of all possible characters. The wildcard characters represent the IUPAC ambiguity codes for nucleic acids to allow limited regular expressions on DNA sequences. The implementation of wildcard characters does not affect the performance of the initial algorithm as the wildcard characters are only processed once during the pre-processing of the tag sequence and not while processing the dataset.

### Detection of fragment-to-fragment concatenations

Fragment-to-fragment concatenations can be identified by subsequences that match to concatenated tag sequences. If two fragments are concatenated, the resulting sequence contains the 3'-end tag of one sequence followed by the 5'-end tag of another sequence (Figure 1). Identifying the concatenated tag sequences inside the sequence reads allows the detection of fragment-to-fragment concatenations and hence separation into the original fragments. The detection of concatenated tag sequences uses a similar approach as for detecting tag sequences at the sequence ends. This allows the user to define a maximum number of mismatches to account for the limitations of the sequencing methods. The user may choose to only allow exact matching for this part of the program to reduce the number of possibly falsely identified approximate tag sequences.

### Automatic tag sequence estimation

**Figure 2:**
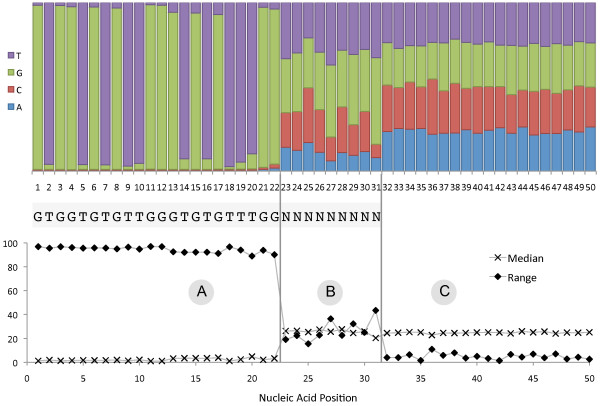
**Example data for the first 50 positions of a metagenomic dataset containing tag sequences**. Example data showing nucleotide frequencies (top), predicted tag sequence (middle), and frequency range and median (bottom) for the first 50 positions in a metagenomic dataset before tag trimming. We can see a clear separation between the non-random nucleotide positions of the tag (A), the quasi-random nucleotides of the tag (B) and the metagenomic sequence (C).

The tag sequences are automatically detected using a nucleotide frequency-based approach. Assume a nucleotide *N_i _*at position *i *has frequency of occurrence  and the sum of all frequencies at position *i *is normalized to 1. If *N_i _*is part of the tag sequence, it should have a frequency  close to 1 and all other nucleotides at position *i *should have a frequency close to 0 (Figure 2A). If *N_i _*is not part of the tag sequence, is should have a frequency  close to 0.25 (1/4 × 1). The 1/4 parameter assumes equal distribution of the A, C, G and T nucleotides in the metagenome (Figure 2C). To account for the non-uniform distribution of nucleotides, it is possible to first estimate the G/C content of the metagenome and adjust the frequencies accordingly. In the current implementation, however, an equal distribution of the nucleotides is assumed and this step omitted. The range and median of the nucleotide frequencies at a position of the tag sequence with a quasi-random nucleotide should show a distinctive pattern from the first two cases with  neither close to 1 or 0 (Figure 2B).

**Figure 3:**
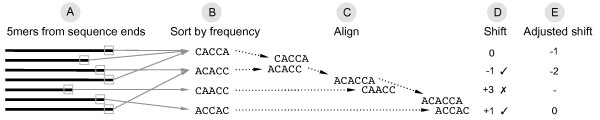
**Simplified example showing the calculation of shift values for 5-mers at the 3'-end**. The 5-mers at the 3'-end of all sequences are extracted (A) and sorted by decreasing frequency (B). The first 5-mer in the list (highest frequency) is then aligned to the second 5-mer (C) to calculate the minimum number of shift operations to align the two 5-mers without gaps (D). The shift direction is based on the 5-mer with the higher frequency. Shifts to the left have negative values assigned, whereas shifts to the right have positive values assigned. If the number of shift operations is less than or equal to a given threshold (default: 2), then the two 5-mers are joined into one *k*-mer. In the next step, the third 5-mer is aligned with either the first 5-mer or the joined *k*-mer and the same operations are performed. These steps are repeated for the remaining 5-mers. The values of shift operations for the 3'-end are then adjusted (E) by the negative of the maximum number of shift operations (*- *max{*-*1, 1}).

The tag sequences might miss nucleotides at the ends (mainly 3'-end) due to the limitations of the sequencing technology. This can cause an overlap of shifted nucleotides of the tag sequence and may result in noisy frequency values. Therefore, nucleotide frequencies are filtered and corrected using *k*-mers before the tag sequence is estimated. The *k*-mers (default: *k *= 5) at the 5'-end and 3'-end of all sequences are extracted and filtered by frequency of occurrence. The *k*-mers that occur in at least 10% of the sequences are sorted by decreasing frequency and all other *k*-mers are rejected. The first *k*-mer in the list (highest frequency) is then aligned to the second *k*-mer to calculate the minimum number of shift operations *l *to align the two *k*-mers without gaps (Figure 3). The shift direction is based on the *k*-mer with the higher frequency. Shifts to the left have negative values assigned (*-l*), whereas shifts to the right have positive values assigned (+*l*). If the second *k*-mer can be aligned by shifts in both directions (e.g. ACACA and CACAC) and min{| - *l*|} = min{| + *l*|}, then the shifts will be assigned ± *l*, otherwise they will be assigned min{| - *l*|, | + *l*|}. If *l *is less than or equal to a given threshold of shift operations (default: 2), then the two *k*-mers are joined into one *k*-mer of length *k *+ *l*. Otherwise, the second *k*-mer is moved to the end of the *k*-mer list. In the next step, the third *k*-mer is aligned with either the first *k*-mer or the joined *k*-mer and the same operations are performed. These steps are repeated until no remaining *k*-mer can be aligned under the described criteria. The values of shift operations are then adjusted by *l *+ *a*, where *a *= |min{*l*}| for the 5'-end and *a *= *- *max{*l*} for the 3'-end. The *k*-mer with the highest frequency has *a *assigned as its adjusted shift value. The frequencies are then shifted for the sequences that contain a *k*-mer with an adjusted shift value. Nucleotide *N*_*i *_is therefore used to calculate .

The difference between range and median of  is used to predict the tag sequence. A frequency range greater than three times the median indicates a specific nucleotide (Figure 2A), whereas a frequency range less than the median plus an allowed variation (default: 5%) indicates nucleotides of the "real" sequence (Figure 2C). The remaining frequencies indicate preferred nucleotides or quasi-random nucleotides (Figure 2B). All continuous nucleotides that fall into the first or last category are defined as tag sequence. This approach works well for sufficiently big datasets (e.g. more than 1,000 reads to detect the quasi-random part of WTA tag sequences; see Additional file 2).

### Implementation and computational platform

The web interface was implemented in Perl 5.8 using the Common Gateway Interface (CGI) module to generate dynamic HTML content, and to input and output data from and to the web interface. The bit-vector algorithm and all other calculations were implemented in Perl 5.8 using dynamic programming methods. The TagCleaner web application is currently running on a PC server with Fedora Linux using an Apache HTTP server to support the web services. The web interface provides a high level of compatibility with heterogeneous computing environments.

It was a design decision to make TagCleaner independent from any third party programs necessary to perform the data processing and analysis. This allows the user to operate TagCleaner on their own servers without the requirement for other software to be installed.

### Input and output

The input for the TagCleaner web interface is FASTA data containing the metagenomic reads. In addition to FASTA files, the user can submit FASTQ files (containing sequence and quality data) [17], which will automatically be converted into FASTA format. The input data is checked to be a valid FASTA or FASTQ file with DNA data. If the input data fails the validation step, further processing is restricted.

Metagenomic sequence files can be of large size (several 100 Mb), and therefore the web interface additionally allows the submission of compressed FASTA or FASTQ files to reduce the time of data upload (by approximately 70%) from the user machine to the web server. The currently supported compression types are ZIP and GZIP. If the compressed files contain more than one FASTA or FASTQ file, the single files will be joined into one dataset. The file formats and compression types are automatically detected and processed accordingly. There is no limit on the number of sequences or the size of the input file accepted by TagCleaner.

In addition to the sequence data, the user can specify a tag sequence. TagCleaner accepts wildcard characters in the form of the IUPAC ambiguity code for nucleic acids (for example Y for C or T). If the tag sequence is not available to the user, the program will try to estimate the tag sequence as described above and then allows the user to modify the tag sequence before further data processing.

The user can download the results in FASTA format or its compressed version. The results can either be the data passing all filters and the tag sequences trimmed, or the data not passing the filters without any changes. This allows the user to further investigate both results separately.

Furthermore, the user can decide if the FASTA output file should include the following additional information in the header line: initial sequence length, sequence length after trimming, 5'-end and 3'-end trimming positions, 5'-end and 3'-end mismatches, and the number of fragments the initial sequence was separated into (see Additional file 3).

Reads that were split and that passed the filter parameters have a counter added to the sequence id in order to allow a valid FASTA format output (containing only unique sequence ids).

Results will be stored for one week, if not otherwise requested, on the web server using a unique identifier displayed on the result page. This identifier allows the user to share the result with other researchers without having to re-submit and re-process the dataset. The filter parameters can be imported and exported to allow consistent analysis of different datasets.

### Summary of filter parameters and system considerations

The user can filter the data based on different parameters. Unlike most other programs, this program allows the user to define filter parameters based on the input data after the data is processed. This does not require an *a priori *knowledge of the best parameters for a given dataset.

The filter parameters include the maximal number of mismatches (or percentage of sequence difference) for tag sequences matching at the 5'-end and 3'-end of the reads, occurrence of tag sequences (5'-end, 3'-end, both ends, either end, or none), and the sequence range from the ends in which the tag sequence has to match. The option to continuously trim tag sequences from the ends allows for trimming of concatenated tag sequences at the ends, and is also used to filter out reads that only consist of tag sequences. Additional parameters are designed for filtering the data after the trimming process. These parameters include minimum and maximum sequence length, removal of exact duplicates, removal of sequences containing the ambiguous base N above a given threshold, and separating fragment-to-fragment concatenated reads. Quality trimming and sequence dereplication is recommend to be performed after tag sequence trimming. The trimming of low-quality bases at the ends might truncate the tag sequence and reduce the ability to recognize the remainder of the tag sequence. In those cases, large parts of the tag sequences might still remain for further analysis and data processing steps. The dereplication before trimming may miss duplicated sequences due to variations in the tag sequences that will be trimmed off later and would therefore require an additional dereplication step after the trimming.

## Results

### Web application

**Figure 4:**
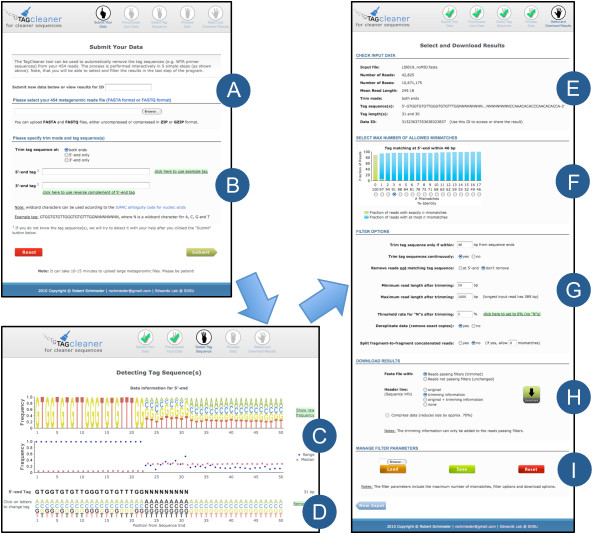
**TagCleaner web interface**. Screenshots of the TagCleaner web interface at different steps of the data processing. The user can either input a data ID to access already processed data (A) or input a new sequence file and the tag sequences, if available (B). If the tag sequence is not available, the tag sequence is estimated using a nucleotide frequency-based approach. The estimated tag sequence is shown below the nucleotide frequency plot and the frequency range and median plot (C). Based on the provided frequency information, the user can change the estimated tag sequence using the functionality of the graphical interface (D). After detecting the tag sequence in the dataset, the results are shown including the input information (E), graphical representation of the number of mismatches (F), filter parameters (G), download options (H) and options to manage filter parameters (I).

TagCleaner is publicly available through a user-friendly web interface (Figure 4). The interactive web interface facilitates navigation through the results, definition of filter parameters, and allows the export of the results for subsequent offline analysis. The input page of TagCleaner provides a mechanism to import new datasets and to define the tag sequence(s). Users can choose between submitting and processing a new dataset or accessing already processed datasets using a unique identifier. The import and export functionality for the filter parameters make it easy for the users to perform the same analysis on different datasets and to record the filter parameters.

### Application examples

In the first application example, TagCleaner was applied to three metagenomic datasets available in FASTA format (Table 2). The datasets were generated as described in Methods and contained tag sequences (WTA primer) at both ends, which needed to be trimmed before further data processing. No prior knowledge of the tag sequences was assumed. The FASTA files were provided as input and the tag trimming for both ends was selected. The tag sequence was in all three cases identified as 5'-GTG GTG TGT TGG GTG TGT TTG GNN NNN NNN N (31 bp) and NNN NNN NNN CCA AAC ACA CCC AAC ACA CCA-3' (30 bp). The results of the tag detection are shown in Table 1. The reverse complement of the 5'-end tag sequence would be expected as tag sequence at the 3'-end. However, the exact reverse complement 5'-end tag sequence (3'-end with additional C) could only be identified in 0.19 - 0.40% of the sequences in the three datasets.

The tag sequence at the 5'-end was identified in 84 - 86% of the sequences without any mismatches, while 96 - 98% of the tag sequences was identified by allowing a maximum of five mismatches. The tag sequence at the 3'-end was identified in 16% of the sequences without allowing any mismatches and in 43 - 56% of the sequences by allowing up to five mismatches. Fragment-to-fragment concatenations were identified in almost 2% of the sequences without any mismatches and in more than 3% of the sequences with up to three mismatches.

Setting the parameters as shown in Table 3 resulted in 34,797 (81.25%), 13,732 (80.17%) and 40,684 (82.55%) sequences passing all filters for LIB019, LIB020 and LIB021, respectively. The majority of the filtered sequences were either shorter than 50 bp, had an occurrence of N above the 5% threshold, or were tag sequence repeats.

**Table 3:** Parameter values used in the first application example

Parameter	Value
Maximum number of mismatches at 5'-end	3

Maximum number of mismatches at 3'-end	3

Sequence range from the ends	46 bp

Continuous trimming	Yes

Remove sequences not matching tag sequence	No ("don't remove")

Minimum read length	50 bp

Maximum read length	Default (maximum length)

Threshold for occurrence of N	5%

Dereplicate data (remove exact sequence copies)	Yes

Fragment-to-fragment splitting	Yes

Maximum number of mismatches for splitting	3

**Figure 5:**
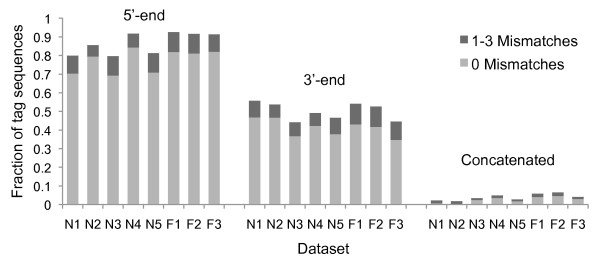
**Results for exact and approximate tag sequence matching for the datasets from Nakamura et al**. [18]. TagCleaner detected the same tag sequences (5'-TGT GTT GGG TGT GTT TGG NNN NNN NNN N and NNN NNN NNN NCC AAA CAC ACC CAA CAC A-3') in the sequences from nasal (F1 - F3) and fecal samples (N1 - N5). The fraction of sequences that contained tag sequences with no mismatches and 1-3 mismatches is shown for the 5'-end, 3'-end and the concatenated tag sequences.

In addition, eight metagenomes from Nakamura et al. [18] were also investigated, as this was the only published dataset still containing WTA tag sequences. The metagenomes were provided in FASTQ files (see Additional file 4). The datasets contained the sequence reads with tag sequences at both ends. The WTA tag sequences were not published in the paper and therefore, automatic tag detection was performed on all FASTQ files. The program detected the same tag sequences (5'-TGT GTT GGG TGT GTT TGG NNN NNN NNN N and NNN NNN NNN NCC AAA CAC ACC CAA CAC A-3') in all datasets. The results for no mismatches and a maximum of three mismatches for the tag sequences are shown in Figure 5. Allowing only exact matches, the datasets contained for more than 2% of the reads concatenated fragments and allowing for a maximum of three mismatches 4% of the sequences were identified as concatenated fragments. A significant number of reads matched to the tag sequences over the whole length (possible tag sequence repeats). These reads were filtered using the continuous trimming of tag sequences from the ends. Further investigation of the fragment-to-fragment concatenated reads was performed using BLASTn against NBCI's non-redundant database. The BLASTn hits were filtered using the same thresholds as described in [18] and taxonomy was assigned to the best hits using the NCBI taxonomy. The nasal samples (F1 - F3) contained more than 90% eukaryotic sequences [18] and showed that concatenated fragments were mainly from the same taxonomic group (see Additional file 5). The fecal sample N3 with more than 80% of the sequences assigned to RNA viruses showed a similar behavior. The four remaining fecal samples (N1, N2, N4 and N5) contained mainly prokaryotic sequences and showed that concatenated fragments were from different taxonomic groups. The number of different BLASTn best hits increased with increasing taxonomic levels.

### Improving assemblies with TagCleaner

The GS De Novo Assembler Software version 2.3 (Roche, Branford, CT) was used to assemble three metagenomic libraries (Table 2) to illustrate how TagCleaner can improve metagenomic and other high-throughput studies. The assembly parameters were set to 95% identity over at least 35 bp. Assemblies were generated for three different parameter sets for each of the metagenomic libraries: (A) raw data; (B) tag sequences trimmed allowing three mismatches; (C) tag sequences trimmed allowing three mismatches with additional splitting of the fragment-to-fragment concatenations and continuous end tag trimming. For B and C, the minimum sequence length was set to 40 bp, sequence duplicates were removed and all other parameters were kept at their default values.

By using TagCleaner, the resulting assemblies showed an increase in the N50 contig size (a standard measure of assembly quality [19-21]) for the datasets assembled with parameters B compared to the raw datasets and an even higher increase using parameters C compared to using the raw data (Table 4). The ratio of number of contigs to number of contigs longer than 500 bp increased for the three datasets from assembly run A to C. Furthermore, the average contig length for all contigs and for contigs longer than 500 bp were also increased.

**Table 4:** Assembly results for three metagenomic datasets

Library	Assembly run	# Reads	# Contigs (> 500 bp)	Average contig length (> 500 bp)	Contig N50^1 ^(bp)	# Concatenated tag sequences allowing 3 mismatches
LIB019	A	42,825	136 (25)	329.91 (703.08)	423	10

	B	34,778	73 (25)	390.04 (694.04)	605	5

	C	35,426^2^	50 (26)	510.94 (768.92)	663	0

LIB020	A	17,129	89 (6)	246.40 (557.33)	306	4

	B	14,208	55 (13)	292.85 (655.85)	510	3

	C	14,366^2^	52 (12)	312.54 (726.33)	547	0

LIB021	A	49,282	305 (15)	238.54 (682.00)	276	29

	B	41,126	186 (18)	264.12 (691.67)	302	16

	C	42,495^2^	165 (20)	282.39 (782.00)	303	0

The GS De Novo Assembler Software version 2.3 (Roche, Branford, CT) was used to assemble three metagenomic libraries (LIB019, LIB020 and LIB021) to illustrate how TagCleaner can improve metagenomic and other high-throughput studies. The assembly parameters were set to 95% identity over at least 35 bp. Assemblies were generated for three different parameter sets for each of the metagenomic libraries: (A) raw data; (B) tag sequences trimmed allowing three mismatches; (C) tag sequences trimmed allowing three mismatches with additional splitting of the fragment-to-fragment concatenations and continuous end tag trimming. For B and C, the minimum sequence length was set to 40 bp, sequence duplicates were removed and all other parameters were kept at their default values.

^1 ^The N50 contig size is a weighted median that is defined as the length of the smallest contig *C *in the sorted list of all contigs where the cumulative length from the largest contig to contig *C *is at least 50% of the total length (sum of contig lengths).

^2 ^Increased number of reads due to splitting of the fragment-to-fragment concatenations.

In all cases for datasets generated with parameter sets A and B, concatenated tag sequences were observed in the contigs, but not for the datasets generated with parameter set C. The contigs with concatenated tag sequences generally showed higher coverage outside the tag sequence regions (see Additional file 6). For further taxonomic analysis, the contigs were split at the concatenated tag sequences and a BLASTn analysis was performed. The separated fragments hit to different taxonomic groups using NCBI's taxonomy assigned to the best hits (data not shown).

### Comparison of TagCleaner with other programs

There are different applications available that are able to trim tag sequences. TagCleaner was compared with five other available programs, each offering various additional features and functions. Although most of the programs have been designed to process 16S tag sequences, they are able to process non-16S sequence data and allow the trimming of their tag sequences. PyroTagger [22] is a program to process and classify multiplexed amplicon pyrosequence data from any region of the 16S rRNA gene. RDP-Pyro [23] is part of the Ribosomal Database Project for the analysis of 16S sequences generated with the pyrosequencing method. SeqTrim [24] is a sequence pre-processing pipeline. SeqClean [25] is a program for trimming and validation of sequences by screening for various contaminants, low quality and low-complexity sequences. Mothur [26] is a software package used to analyze community sequence data. It incorporated programs such as DOTUR and SONS and contains modules to trim tag sequences. In Table 5, we have compared TagCleaner with these programs for features related to tag trimming.

**Table 5:** Comparison of TagCleaner with other applications performing tag trimming

	TagCleaner	PyroTagger	RDP-Pyro	SeqTrim	SeqClean	Mothur
Web-based	Yes	Yes	Yes	Yes	-	-

Standalone	-	Yes	-	Yes	Yes	Yes

Trim both ends	Yes	-^1^	Yes	Yes	Yes	Yes

Provide trimming information^2^	Yes	-	Yes	Yes	Yes	-

Support IUPAC ambiguity codes	Yes	Yes	Yes	-	-	Yes

# Allowed errors	Any	0	0, 1, 2	Any^3^	Any^3^	0

Predict tag sequence	Yes	-	-	-	-	-

Detect fragment- to-fragment con- catenations	Yes	-	-	-	-	-

Continuous trimming^4^	Yes	-	-	-	-	-

The comparison is based on the features related to tag trimming, as this represents the main purpose of TagCleaner. All compared applications are still in active development and new functions will undoubtedly be added over time.

^1 ^Performs barcode trimming at 5'-ends only.

^2 ^Trimming position(s) and/or number of trimmed bases.

^3 ^Percent similarity between tag sequence and query sequence (BLAST-based comparison).

^4 ^Trimming of tag sequence repeats

## Discussion

Tag sequence contaminations are a serious concern to the quality of the data used for downstream analysis. Therefore, it is important to use reliable tools for the pre-processing of sequence data. We presented a web-based program that implements several features to improve the pre-processing of the data.

The assemblies of our in-house dataset showed the improvement of the pre-processed data. The results show a particularly good example of the need for allowing mismatches in the tag sequence and for identifying fragment-to-fragment concatenations.

The algorithm of Myers has superior performance compared to other algorithms when applied to biological sequence data [8], but is bounded by the architecture of the system used. Systems with 32 or 64 bit architectures basically allow tag sequences of at most 32 or 64 nucleotides, respectively. However, longer tag sequences can be handled using Perl modules for bit-vector representation. The current implementation does not make use of those modules since they will reduce the efficiency of the program. Furthermore, very long tag sequences, especially primer sequences with more than 64 bp are rarely used for high-throughput sequencing.

The algorithm implemented in TagCleaner for the automatic detection of tag sequences assumes the randomness of a typical metagenome. Datasets that do not contain random sequences from organisms in an environment, but rather contain, for example, 16S metagenomes may cause incorrect detection of the tag sequences. However, the tag sequences will most likely be over-predicted and can be redefined by the user prior to data processing.

There are several advantages in using TagCleaner to pre-process sequence data: tag sequence trimming data filtering improve the reliability of downstream data analysis; TagCleaner is a web application that allows users to pre-process their datasets without installing any software; TagCleaner is independent of third-party software and thus compatible with any computer supporting web services.

To our knowledge, TagCleaner is the first web application optimized to automatically detect and remove tag sequences from metagenomic datasets. Furthermore, no other freely available web application or standalone tool implements the additional feature of detecting and splitting fragment-to-fragment concatenations. This important pre-processing step removes tag contaminations inside the sequences, which may allow, for example, more accurate assemblies. The concatenated fragments may additionally present a source of error for annotation and taxonomic assignments, since fragments from different organisms may not be assigned correctly when concatenated. The continuous trimming of tag sequences from the ends allows filtering of sequences mainly consisting of concatenated tag sequences.

TagCleaner does not require the setting of filter parameters (such as maximum number of mismatches) before the data is processed. Instead, the filter parameters are set after the data is processed, which allows the user to choose parameters appropriate for their dataset and does not require them to submit and process the same data with modified parameters for several times.

The independent parameter definition for tag sequences matching at the 5'-end and 3'-end of the reads accounts for the differences in tag sequences due to the limitations of the sequencing method used to generate the datasets.

The ambiguous code extension represents another advantage over other programs that are, for example, based on BLAST comparisons and do not allow the use of ambiguous letters. BLAST is not able to perform a search on sequences that contain ambiguous bases. This means that BLAST-based programs either need to search for all possible combinations or are not able to match the ambiguous positions. Furthermore, BLAST implements a heuristic that might not allow the correct identification of all tag sequences, whereas the bit-vector algorithm implemented in TagCleaner is able to return the correct positions of matching tag sequences.

TagCleaner is also able to detect the quasi-random 3'-end of WTA primers. The user has the option whether or not to trim this part of the tag sequence by simply adding or removing the letter N from the end of the tag sequence. However, we do advise users to trim the complete tag sequence. It is important to trim the random parts in order to account for mismatch-induced mutations that often happen when primers anneal to similar (but not identical) sequences with high enough affinity for binding (see Additional file 7). Therefore, we cannot be certain that this part of the tag sequence represents the actual sequence of the sample.

TagCleaner can be used to trim tag sequences from both ends or from a single end. This allows the trimming of MID tags from the 5'-end that are exact matches, or approximate matches by allowing mismatches.

The additional filter option provided by TagCleaner include the removal of short sequences and sequences containing the ambiguous base N. Excluding those sequences can reduce the error rate of the data set. Huse et al. showed that the presence of the ambiguous base N was an effective indicator of a low-quality sequence and additionally suggest that shorter sequences (e.g. trimmed by sequencing software for bases presumed to be in error) are more likely to be of dubious quality [15].

The high error rate in the WTA tag sequences (Table 1 and Additional file 4) reflects the limitations of the pyrosequencing approach. We did not see such a high error rate for MID tags that were optimized for 454 pyrosequencing, suggesting that Transplex WTA tag sequences do provide a source for higher error rates due to the GT pattern.

## Conclusions

This new web-application, TagCleaner, provides scientists with the means to automatically detect and remove tag sequences from metagenomic reads without prior knowledge about the sequencing protocol, thereby enabling the analysis of public data still containing tag sequences. If the tag sequence is known, TagCleaner still provides an efficient and effective alternative to other tag removal programs by providing additional filter options such as removing short reads and duplicated reads, as well as separating reads that were a result of fragment concatenations prior to sequencing.

TagCleaner's interface is simple and user-friendly. Additionally, since TagCleaner is a web application and independent from third party programs such as BLAST, both large and small research laboratories can easily use it. TagCleaner allows users of small research laboratories, which use external applications or pipelines that are not able to remove tag sequence sufficiently, to pre-process and filter their data and continue using the external applications for downstream analysis.

## Availability and requirements

• **Project name: **TagCleaner

• **Project home page: **http://tagcleaner.sourceforge.net

• **Operating system(s): **Web service, platform independent

• **Programming language: **Perl

• **Restrictions to use by non-academics: **None

## Authors' contributions

RS has designed and implemented the web application, tested the program, and drafted the initial manuscript. YWL processed the samples, prepared the metagenomic libraries, and participated in the GUI component design and testing. FR and RE conceived of the study, and participated in its design and coordination. All authors read and approved the final version of the manuscript.

## Supplementary Material

Additional file 1 **Fragment length distribution**. Length distributions are shown as estimated by the Agilent 2100 Bioanalyzer (Agilent Technologies, Inc., Santa Clara, CA) for the libraries LIB019, LIB020 and LIB0021. Click here for file

Additional file 2 **Nucleotide frequency logos showing the difference between raw and filtered nucleotide frequencies**. Nucleotide frequency logos showing the raw frequencies (top) and the filtered and corrected frequencies (bottom). Both logos are provided to the user on the web interface to support the detected tag sequence. The tag sequence at the 3'-end can be identified more easily in the bottom logo and therefore allows more accurate predictions. (The lower value for nucleotide A at position 1 in the bottom logo shows that the majority of the sequences are shifted to the left by one position.) Click here for file

Additional file 3 **Example sequence explaining the modified header line as generated by TagCleaner**. Click here for file

Additional file 4 **Results for exact and approximate tag sequence matching on the datasets from Nakamura et al**. [18]. The maximum allowed mismatches are abbreviated by MM. Percentages are shown in parenthesis. Click here for file

Additional file 5 **BLASTn results for concatenated fragments found in the datasets from Nakamura et al**. [18]. BLASTn was performed against NCBI's non-redundant database and taxonomy was assigned according to the NCBI taxonomy. BLAST hits had to have an E-value of less than 10^-5 ^and the best hits were used to calculate the fractions. Click here for file

Additional file 6 **Contig coverage plots**. Bars mark the locations of the concatenated tag sequences. Click here for file

Additional file 7 **Example of imperfect primer annealing that causes mismatch-induced mutations in the sequence reads**. Click here for file
