# Supplementary figures and images for: TagCleaner: Identification and removal of tag sequences from genomic and metagenomic datasets
Source: BMC Bioinformatics. 2010 Jun 23;11:341. doi: 10.1186/1471-2105-11-341 (PMC2910026; doi:10.1186/1471-2105-11-341)

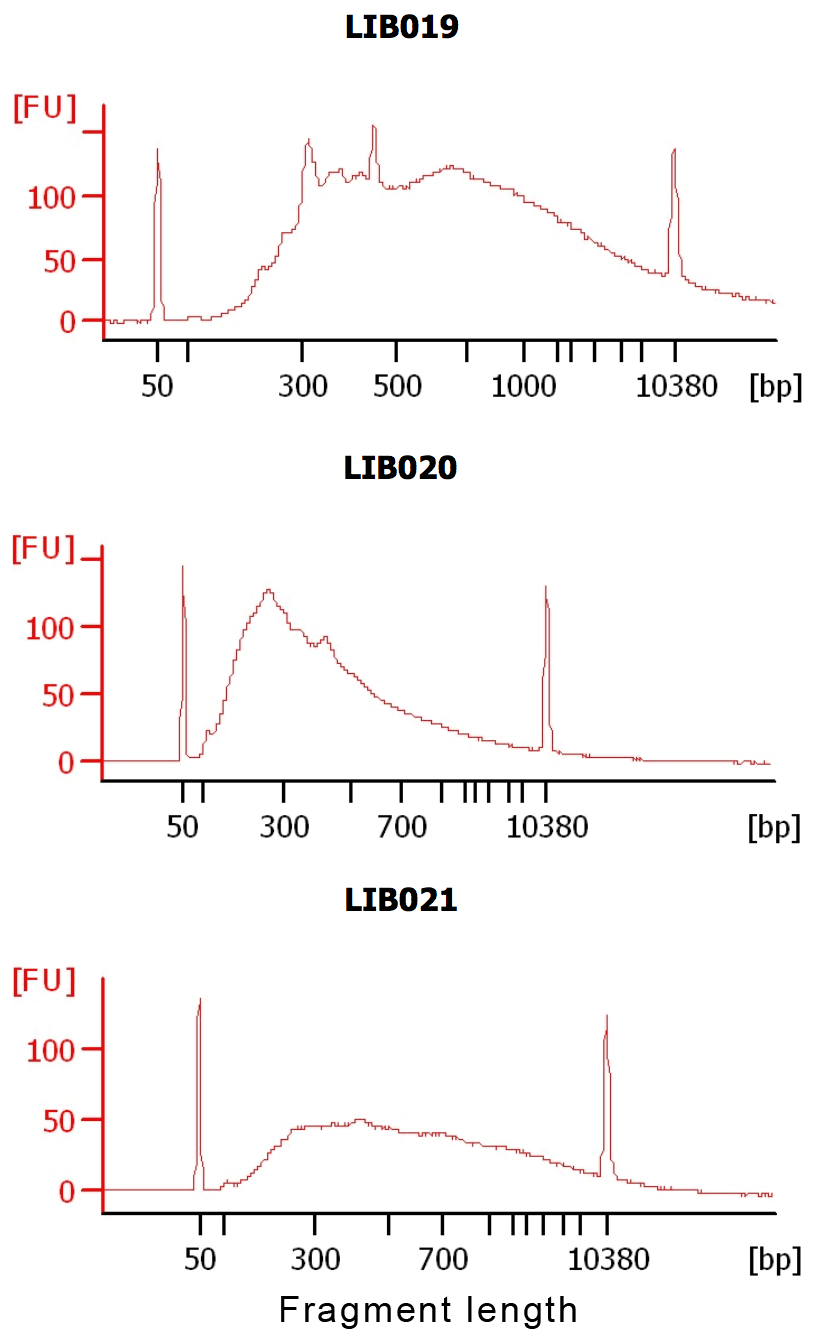

Supplement: Additional file 1 — Fragment length distribution. Length distributions are shown as estimated by the Agilent 2100 Bioanalyzer (Agilent Technologies, Inc., Santa Clara, CA) for the libraries LIB019, LIB020 and LIB0021. [file 1471-2105-11-341-S1.PNG]

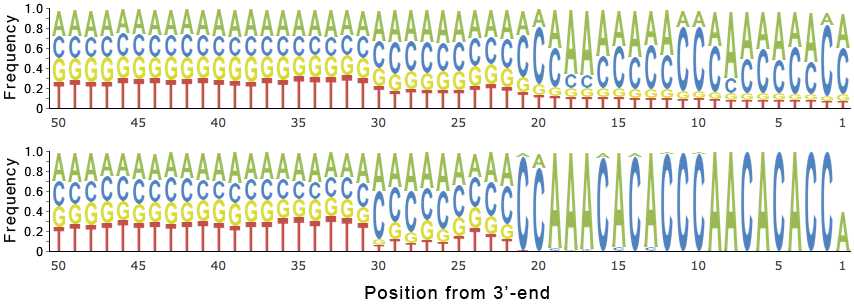

Supplement: Additional file 2 — Nucleotide frequency logos showing the difference between raw and filtered nucleotide frequencies. Nucleotide frequency logos showing the raw frequencies (top) and the filtered and corrected frequencies (bottom). Both logos are provided to the user on the web interface to support the detected tag sequence. The tag sequence at the 3'-end can be identified more easily in the bottom logo and therefore allows more accurate predictions. (The lower value for nucleotide A at position 1 in the bottom logo shows that the majority of the sequences are shifted to the left by one position.) [file 1471-2105-11-341-S2.PNG]

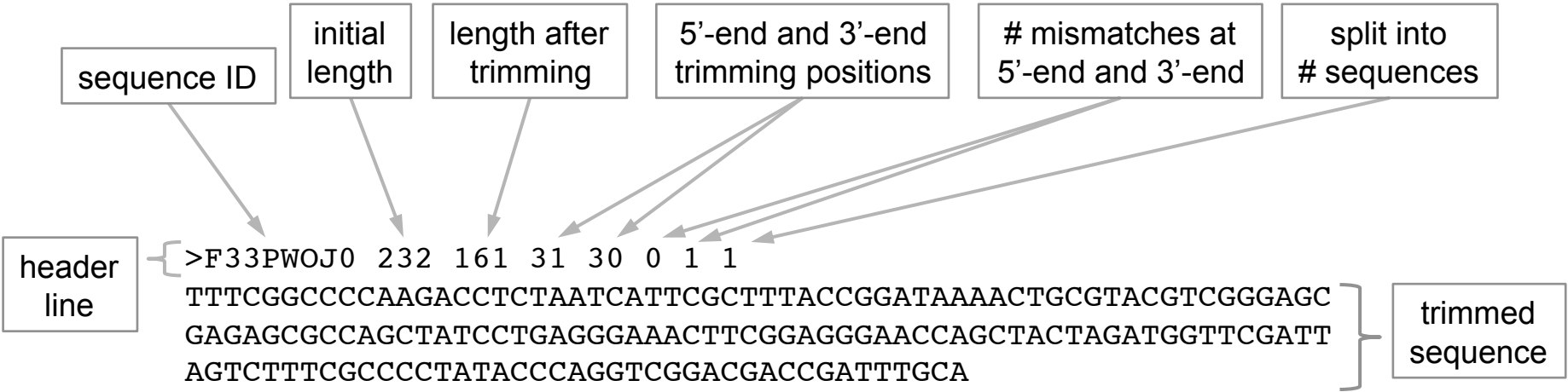

Supplement: Additional file 3 — Example sequence explaining the modified header line as generated by TagCleaner. [file 1471-2105-11-341-S3.PDF]

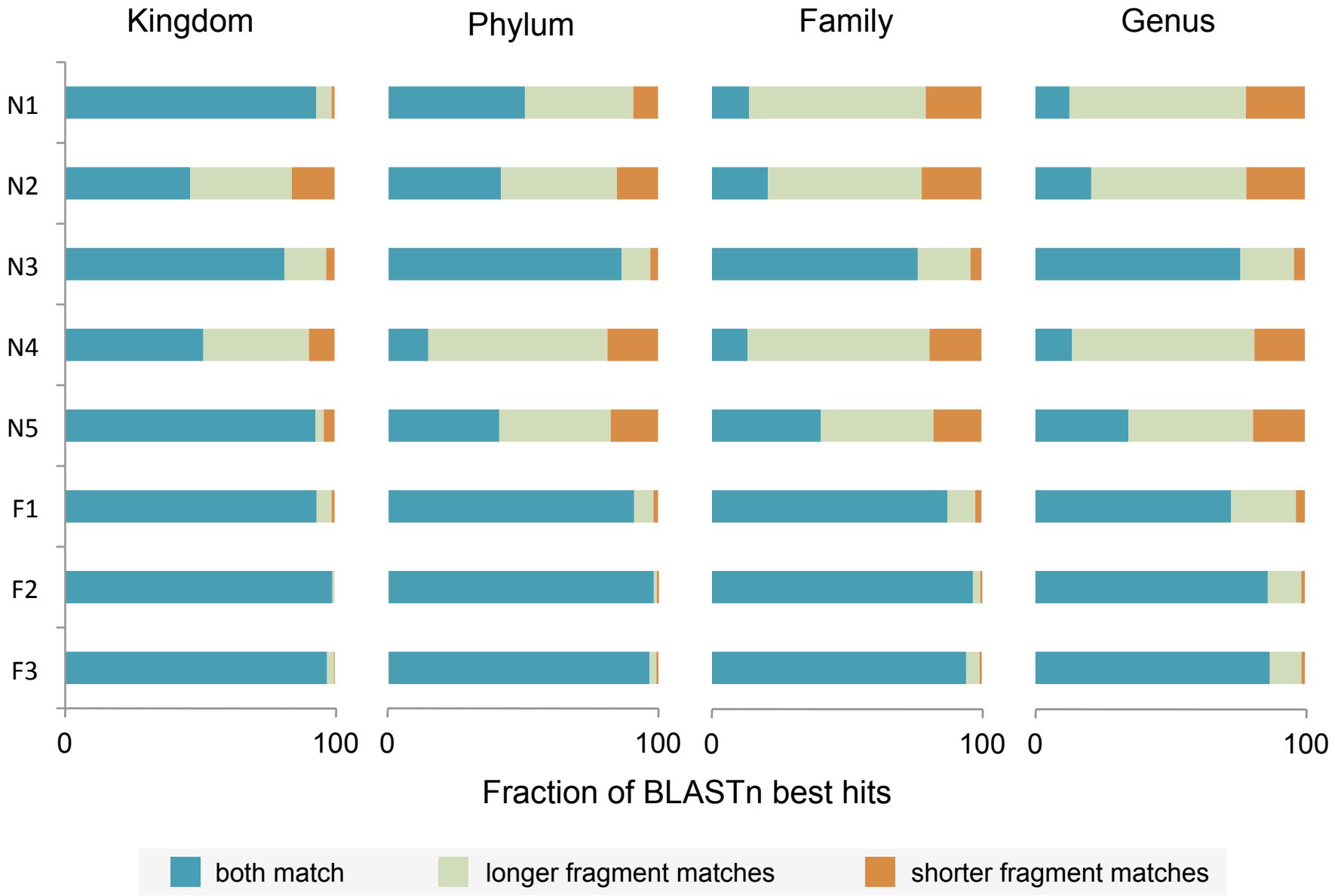

Supplement: Additional file 5 — BLASTn results for concatenated fragments found in the datasets from Nakamura et al. [18]. BLASTn was performed against NCBI's non-redundant database and taxonomy was assigned according to the NCBI taxonomy. BLAST hits had to have an E-value of less than 10-5 and the best hits were used to calculate the fractions. [file 1471-2105-11-341-S5.PDF]

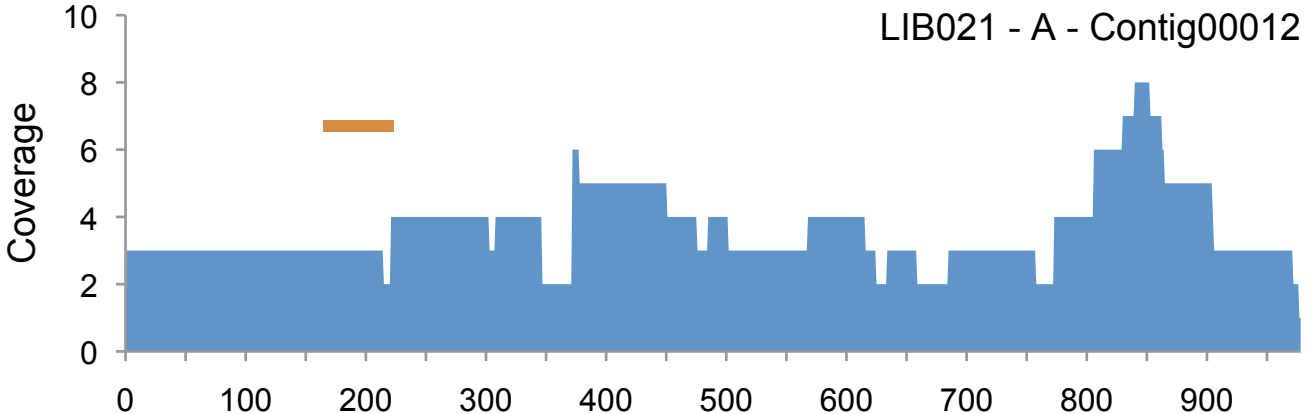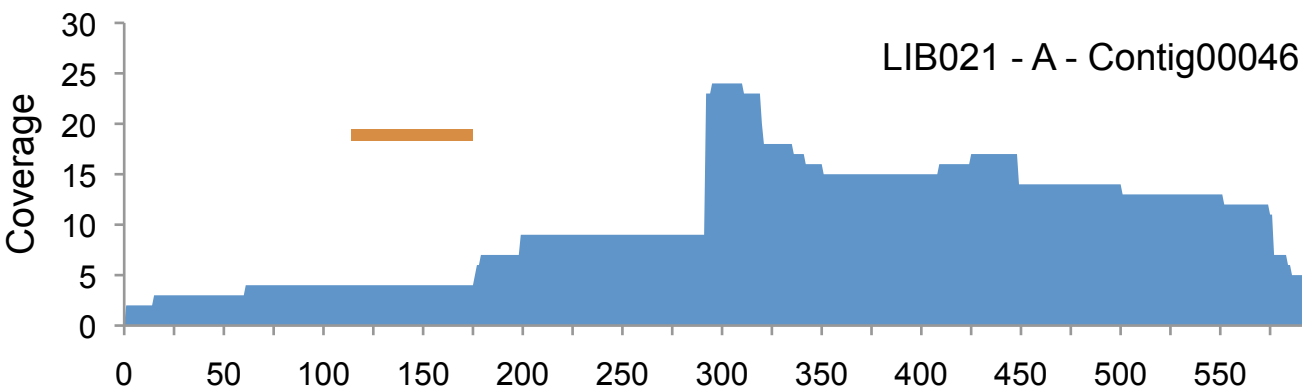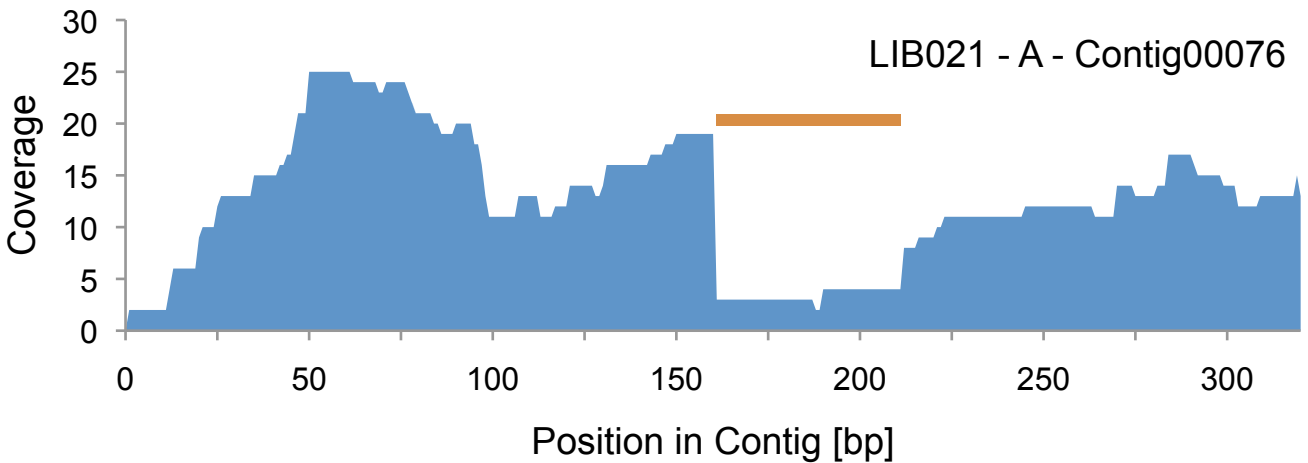

Supplement: Additional file 6 — Contig coverage plots. Bars mark the locations of the concatenated tag sequences. [file 1471-2105-11-341-S6.PDF]
