# Supplementary material for: TagCleaner: Identification and removal of tag sequences from genomic and metagenomic datasets
Source: BMC Bioinformatics. 2010 Jun 23;11:341. doi: 10.1186/1471-2105-11-341 (PMC2910026; doi:10.1186/1471-2105-11-341)
Supplement: Additional file 4 — Results for exact and approximate tag sequence matching on the datasets from Nakamura et al. [18]. The maximum allowed mismatches are abbreviated by MM. Percentages are shown in parenthesis. [file 1471-2105-11-341-S4.PDF]

Filename	Sample	# Reads	# Bases	Mean Length	5'-end		3'-end		Concatenated	
					0 MM	3 MM	0 MM	3 MM	0 MM	3 MM
SRR004254.fastq	N1	15,298	3,122,243	204.09	10,752 (70.28)	12,239 (80.00)	7,167 (46.85)	8,527 (55.74)	95 (0.62)	360 (2.35)
SRR004255.fastq	N2	32,334	6,832,282	211.30	25,691 (79.46)	27,716 (85.72)	15,078 (46.63)	17,377 (53.74)	64 (0.20)	610 (1.89)
SRR004256.fastq	N3	25,500	5,141,378	201.62	17,660 (69.25)	20,332 (79.73)	9,350 (36.67)	11,298 (44.31)	616 (2.42)	885 (3.47)
SRR004257.fastq	N4	18,013	3,776,558	209.66	15,192 (84.34)	16,545 (91.85)	7,605 (42.22)	8,877 (49.28)	634 (3.52)	889 (4.94)
SRR004258.fastq	N5	28,823	6,100,885	211.67	20,417 (70.84)	23,445 (81.34)	10,902 (37.82)	13,452 (46.67)	513 (1.78)	818 (2.84)
SRR004259.fastq	F1	30,958	6,227,863	201.17	25,357 (81.91)	28,644 (92.53)	13,334 (43.07)	16,778 (54.20)	1,257 (4.06)	1,820 (5.88)
SRR004260.fastq	F2	25,119	4,972,122	197.94	20,379 (81.13)	23,056 (91.79)	10,460 (41.64)	13,260 (52.79)	1,140 (4.54)	1,628 (6.48)
SRR004261.fastq	F3	21,858	4,384,650	200.60	17,926 (82.01)	19,991 (91.46)	7,597 (34.76)	9,771 (44.70)	639 (2.92)	915 (4.19)
**Mean %**					**77.40**	**86.80**	**42.21**	**50.18**	**2.51**	**4.00**
