# Supplementary material for: TagCleaner: Identification and removal of tag sequences from genomic and metagenomic datasets
Source: BMC Bioinformatics. 2010 Jun 23;11:341. doi: 10.1186/1471-2105-11-341 (PMC2910026; doi:10.1186/1471-2105-11-341)
Supplement: Additional file 7 — Example of imperfect primer annealing that causes mismatch-induced mutations in the sequence reads. [file 1471-2105-11-341-S7.PDF]

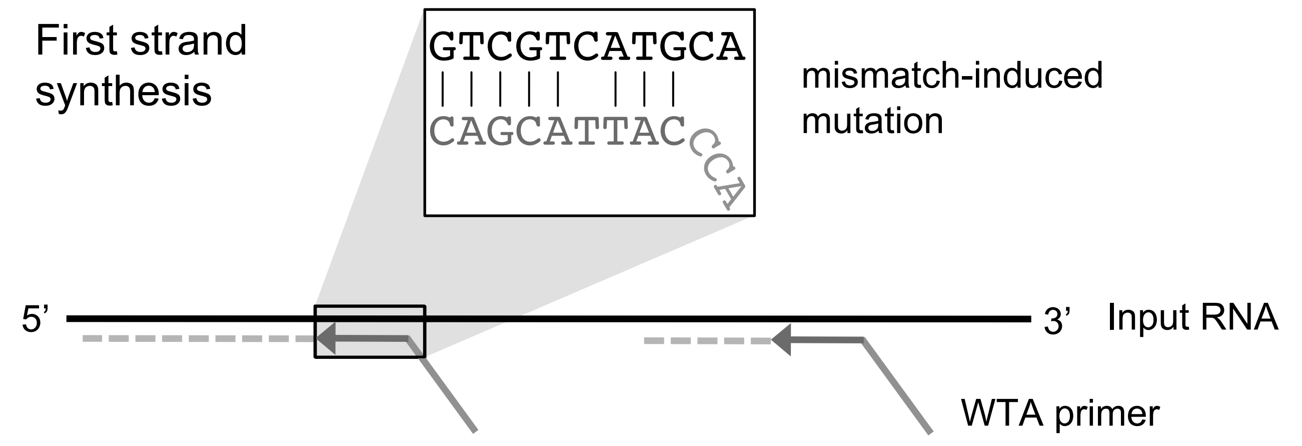
First strand synthesis
GTCGTCATGCA
CAGCATTAC
CCA
mismatch-induced mutation
5'
3'
Input RNA
WTA primer
